# Type 2 Diabetes Is Associated with a Different Pattern of Serum Polyamines: A Case–Control Study from the PREDIMED-Plus Trial

**DOI:** 10.3390/jcm8010071

**Published:** 2019-01-10

**Authors:** Jose C. Fernandez-Garcia, Antoni Delpino-Rius, Iris Samarra, Daniel Castellano-Castillo, Araceli Muñoz-Garach, Maria R. Bernal-Lopez, Maria I. Queipo-Ortuño, Fernando Cardona, Bruno Ramos-Molina, Francisco J. Tinahones

**Affiliations:** 1 Department of Endocrinology and Nutrition, Virgen de la Victoria University Hospital, Institute of Biomedical Research in Malaga (IBIMA), Malaga 29010, Spain; josecarlosfdezgarcia@hotmail.com (J.C.F.-G.); danie__cc@hotmail.com (D.C.-C.); aracelimugar@gmail.com (A.M.-G.); maribelqo@gmail.com (M.I.Q.-O.); fjtinahones@hotmail.com (F.J.T.); 2 CIBER Physiopathology of Obesity and Nutrition (CIBERobn), Institute of Health Carlos III, Madrid 28029, Spain; robelopajiju@yahoo.es; 3 Eurecat, Centre Tecnològic de Catalunya, Centre for Omic Sciences (Joint Unit Eurecat-Universitat Rovira i Virgili), Unique Scientific and Technical Infrastructure (ICTS), Reus 43204, Spain; antoni.delpino@eurecat.org (A.D.-R.); iris.samarra@urv.cat (I.S.); 4 Department of Internal Medicine, Regional University Hospital of Malaga, Institute of Biomedical Research in Malaga (IBIMA), University of Malaga, Malaga 29010, Spain; 5 Department of Medical Oncology, Virgen de la Victoria University Hospital, Institute of Biomedical Research in Malaga (IBIMA), Malaga 29010, Spain

**Keywords:** polyamines, diabetes, insulin, HbA1c, case-control

## Abstract

**Objective:** Polyamines are naturally occurring cationic molecules present in all living cells. Dysregulation of circulating polyamines has been reported in several conditions, but little is known about the levels of serum polyamines in chronic metabolic disorders such as type 2 diabetes (T2D). Therefore, the aim of this study was to evaluate the polyamine-related metabolome in a cohort of metabolic syndrome individuals with and without T2D. **Design and methods:** This was a nested case–control study within the PREDIMED-Plus trial that included 44 patients with T2D and 70 patients without T2D. We measured serum levels of arginine, ornithine, polyamines, and acetyl polyamines with an ultra-high performance liquid chromatography tandem mass spectrometry platform. **Results:** Our results showed that serum putrescine, directly generated from ornithine by the catalytic action of the biosynthetic enzyme ornithine decarboxylase, was significantly elevated in patients with T2D compared to those without T2D, and that it significantly correlated with the levels of glycosylated hemoglobin (HbA1c). Correlation analysis revealed a significantly positive association between fasting insulin levels and spermine. Multiple logistic regression analysis (adjusted for age, gender and body weight index) revealed that serum putrescine and spermine levels were associated with a higher risk of T2D. **Conclusions:** Our study suggests that polyamine metabolism is dysregulated in T2D, and that serum levels of putrescine and spermine are associated with glycemic control and circulating insulin levels, respectively.

## 1. Introduction

Polyamines, cationic compounds widely distributed in living organisms, participate in a myriad of cellular functions, including cell proliferation, apoptosis, and differentiation [1,2,3]. Putrescine, spermidine, and spermine are the predominant polyamines in mammalian cells. Polyamine levels are tightly regulated by a fine-tuned mechanism involving biosynthesis, catabolism, degradation and transport [3,4,5]. However, polyamine metabolism can be dysregulated in certain pathological conditions such as cancer, inflammation, stroke, neurological disorders and renal failure [5,6,7,8]. In transgenic mouse models, the dysregulation of polyamine metabolism has been shown to impact glucose, lipid and energy homeostasis [9,10,11,12,13,14]. In addition, a number of studies using animal models of obesity have reported impaired levels of polyamines in adipose tissue [15], liver [16,17], pancreatic islets [18], and urine [19].

Emerging evidence suggests that increased levels of polyamines in white adipose tissue, liver or skeletal muscle could stimulate energy expenditure and confer resistance to diet-induced obesity and non-alcoholic fatty liver disease [12,13]. Remarkably, polyamine metabolism has been directly implicated in adipogenesis [20,21,22], suggesting that increased polyamine levels might be involved in adipose tissue expansion in obesity. In diet-induced obesity mouse models, a high-dose daily administration of either spermidine or spermine has been shown to be an effective strategy for weight loss and improvement of glycemic status [23,24,25], indicating a possible therapeutic role for exogenous polyamines in metabolic disorders. In addition, emerging evidence has suggested a beneficial effect of chronic low-dose oral spermidine administration on cardiovascular disease in hypertensive experimental models [26]. In humans, epidemiological studies have shown association of a high consumption of dietary spermidine with a reduction in cardiovascular events and decreased mortality [26,27].

While the above discussion indicates that increased polyamine levels may be clinically beneficial, several studies support a contrary conclusion. For example, polyamine levels in human adipose tissue are elevated in insulin-resistant morbidly obese subjects as compared to their insulin-sensitive counterparts [28]. Furthermore, blood polyamines have been reported to be significantly higher in obese subjects as compared with normal weight controls [29]. However, the role of polyamines in these pathological conditions and in type 2 diabetes (T2D), frequently associated with obesity and insulin resistance, is not fully understood. In the pancreas, polyamines are mainly located within the secretory granules of the islet β cells [30], where they have been implicated in proinsulin biosynthesis and insulin secretion [31]. Islet polyamine levels diminish with both age and obesity [18], suggesting that alterations in the intracellular levels of polyamines might affect β cell function. Remarkably, transgenic mice which overexpress the polyamine catabolic enzyme spermidine/spermine acetyltransferase (SSAT), which contains higher levels of putrescine and reduced levels of spermidine and spermine in the pancreatic islets, display impaired glucose-stimulated insulin secretion [11].

Taking these various studies together, it is clear that further information is needed to confirm the association of polyamines with diseases such as diabetes and obesity. The aim of this study was to determine whether serum levels of polyamines are indeed positively associated with T2D and with the degree of glycemic control in a cohort of subjects with metabolic syndrome.

## 2. Materials and Methods

### 2.1. Study Design and Participants

This is a cross-sectional analysis of 114 subjects (>55 years) included in the PREDIMED-Plus trial (clinical trial registration number 89898870) [32], who were consecutively recruited at the Virgen de la Victoria University Hospital (Malaga, Spain). All study subjects met at least three components of the metabolic syndrome (updated harmonized criteria of the International Diabetes Federation and the American Heart Association and National Heart, Lung and Blood Institute) [33], and all were overweight or obese (body mass index, BMI 27–40 kg/m^2^).

Out of these 114 subjects, 44 participants had T2D and 70 participants were non-diabetic. T2D was defined by HbA1c levels ≥ 6.5% or fasting plasma glucose ≥ 126 mg/dL according to the American Diabetes Association (ADA) guidelines [34]. All participants provided written informed consent, and the study protocol and procedures were approved according to the ethical standards of the Declaration of Helsinki by the Research Ethics Committees from all the participating institutions.

### 2.2. Laboratory Measurements

Blood samples were obtained from the antecubital vein after an overnight fast and placed in vacutainer tubes after an overnight fast. Excessive agitation of blood specimens was avoided to minimize hemolysis and serum was immediately separated from the blood samples by centrifugation for 10 min at 4000 rpm and frozen at −80 °C until analysis. Enzymatic methods (Randox Laboratories Ltd., Crumlin, UK) were employed to analyze the levels of serum cholesterol, triglycerides, HDL-cholesterol, glucose, and glycosylated hemoglobin (HbA1c) using a Dimension Vista autoanalyzer (Siemens Healthcare Diagnostics, Erlangen, Germany). Serum insulin levels were measured by immunoassay using an ADVIA Centaur autoanalyzer (Siemens Healthcare Diagnostics). Insulin resistance (IR) was calculated from the homeostasis model assessment of IR (HOMA-IR) with the formula: HOMA-IR = [fasting serum insulin (μU/mL) × fasting blood glucose (mmol/L)]/22.5.

### 2.3. Sample Preparation and Ultra-High Performance Liquid Chromatography Tandem Mass Spectrometry (UHPLC-MS/MS) Analysis of Serum Polyamine Levels

Fifty microliters of serum were aliquoted to a 1.5 mL Eppendorf LoBind tube and mixed with 5 μL of internal standard and 167 μL of methanol. Protein in the samples was precipitated by vortexing for 1 min. A volume of 334 μL of chloroform was added and the mixture was vortexed for 1 min and centrifuged for 10 min at 15,000 rpm and 4 °C. The upper layer was collected and transferred to a tube. In order to derivatize the sample, 100 μL of carbonate-bicarbonate buffer (pH 9) and 50 μL of dansyl chloride (10 mg/mL in acetone) were added. The mixture was vortexed and placed in the dark for 1 h at room temperature. The compounds were extracted with 250 μL of ethyl acetate twice; prior to the second extraction, 2.5 μL of trifluoroacetic acid were added. The combined organic phases were evaporated in a SpeedVac at 45 °C and stored at −20 °C until analysis. Samples were reconstituted in 50 μL of ammonium acetate 0.2 M:acetonitrile (30:70).

Quantification was performed with the commercial standards arginine, ornithine, N^1^-acetylspermidine, N^8^-acetylspermidine, N-acetylputrescine, N-acetylspermine, spermine, spermidine, putrescine, N^1^,N^12^-diacetylspermine, and N^1^,N^8^-diacetylspermidine (Toronto Research Chemicals, North York, ON, Canada). Amino acid internal standards were lysine (^13^C_6_, ^15^N_2_) and arginine (^13^C_6_, ^15^N_4_) (Cambridge Isotope Laboratories) and for polyamines spermine-d20, spermidine-d6, putrescine-d8 and N^8^-acetylspermidine-d3 (Toronto Research Chemicals).

Chromatography was performed with an Agilent UHPLC 1290 series binary pump (Agilent Technologies, Santa Clara, CA, USA), and separation was carried out on a Kinetex EVO C18 column (2.6 µm particle size, 2.1 mm internal diameter × 150 mm length) (Phenomenex, Torrance, CA, USA) held at 25 °C. The mobile phase for elution was a gradient established between water acidified with 0.1% formic acid (A) and acetonitrile acidified with 0.1% formic acid (B) at a flow rate of 400 µL/min. The injected amount was 2.5 µL.

MS/MS analysis was performed on an Agilent QqQ 6490 Series mass spectrometer operating in AJS + ESI. The ionization source parameters were optimized using MassHunter Optimizer (Agilent Technologies, version 6.0) as follows: nebulizer gas (nitrogen) with a pressure of 15 psi, a gas flow of 15 L/min at 200 °C, a sheath gas flow of 11 L/min at 350 °C, a capillary voltage of 2.5 kV, and a nozzle voltage of 1000 V.

### 2.4. Statistical Analysis

Results are given as the mean ± SD. To test normality of the study variables, a Kolmogorov–Smirnov test was applied. Comparisons between study groups of the parameters with and without a normal distribution were performed using Student’s *t*-test and Mann–Whitney *U* test, respectively. Spearman correlation analyses were performed to determine associations between serum polyamine levels with several anthropometric and biochemical variables. Multiple linear regression analysis was performed to determine the associations between HbA1c and HOMA-IR with clinical variables and serum polyamines. A multiple logistic regression model was used to estimate odds ratios (OR) and 95% confidence intervals (CI) of gender, age, BMI, and serum putrescine levels, with T2D as dependent variable. All statistical analyses were performed with SPSS version 22.0 for Windows (SPSS Iberica, Madrid, Spain). *p* < 0.05 was considered as statistically significant.

## 3. Results

**Figure 1:**
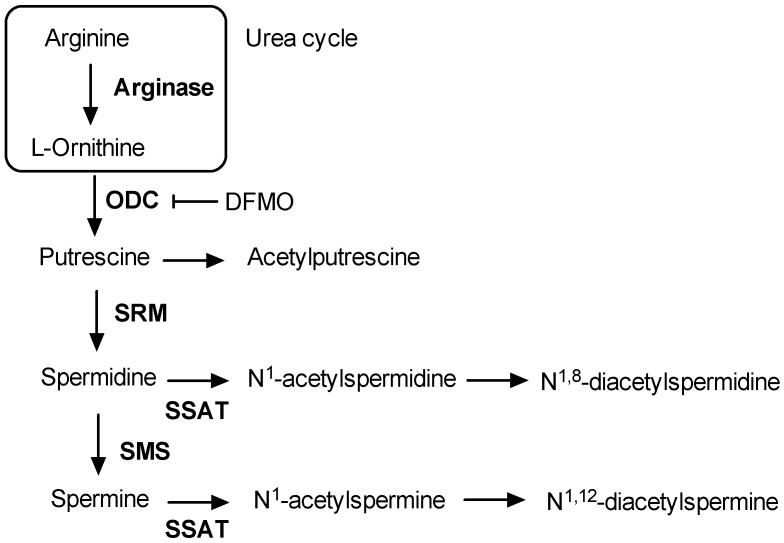
A simplified scheme of polyamine metabolic pathway in eukaryotic cells. DFMO: difluoromethylornithine; ODC: ornithine decarboxylase; SMS: spermine synthase; SRM: spermidine synthase; SSAT: spermidine/spermine acetyltransferase.

Clinical characteristics in patients with T2D and without T2D are shown in Table 1. While there were no significant differences in age, blood pressure, HDL-cholesterol or triglyceride levels between study groups, patients with T2D showed significantly higher BMI, waist circumference, HbA1c levels, fasting glucose, insulin, and HOMA-IR. Among the measured polyamine-related metabolites, only arginine, ornithine, and putrescine were significantly different between T2D and non-diabetic subjects (Table 2). Whereas the serum levels of arginine and ornithine were lower in T2D subjects, putrescine, which is directly produced from ornithine by the enzymatic activity of ornithine decarboxylase (ODC) (Figure 1), was increased. No significant differences in the serum levels of other polyamines such as spermidine and spermine, or their acetylated derivatives acetylputrescine, N^1^-acetylspermidine, N^8^-acetylspermidine, N^1^,N^8^-diacetylspermidine, N^1^-acetylspermine, and N^1^,N^12^-diacetylspermine were detected in diabetic patients as compared to those without T2D.

**Figure 2:**
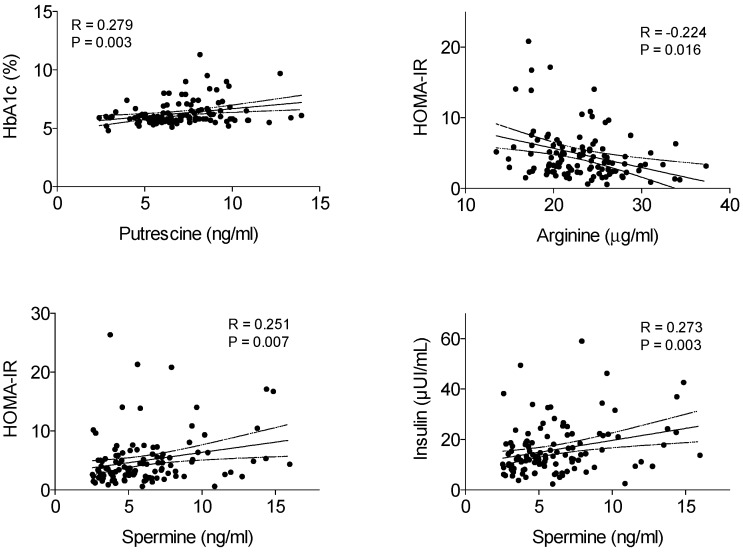
Spearman correlations of serum arginine or polyamine (putrescine and spermine) levels with clinical parameters related to T2D.

Spearman correlation analysis revealed a significant positive correlation between HbA1c and serum putrescine levels (Figure 2). However, no correlation between putrescine, fasting glucose or other clinical parameters such as BMI or HOMA-IR was observed. In addition, serum levels of arginine were negatively correlated with HOMA-IR, whereas there was a significant positive association between serum spermine levels and HOMA-IR (Figure 2). Interestingly, spermine significantly correlated with insulin levels (Figure 2) but not with glucose levels (the two variables used to calculate HOMA-IR index). No significant correlation was observed between BMI and the serum levels of polyamines or related metabolites. Multiple linear regression analysis, performed after adjusting for age and gender, showed a significant positive association between HbA1c and putrescine (Table 3), and between HOMA-IR and spermine (Table 4). Further multiple logistic regression analysis (after adjusting for age, gender, and BMI) revealed that both putrescine and spermine levels were associated with a higher risk of T2D (Table 5). Association between putrescine and T2D were still found when fasting insulin was included in the model (Supplementary Table S1). However, no association between serum polyamines and T2D was observed when either HOMA-IR or fasting glucose was added in the logistic model (Supplementary Table S1).

## 4. Discussion

In this study we have investigated the circulating profile of metabolites related to polyamine metabolism in a cohort of metabolic syndrome patients with and without T2D. Analysis of the serum polyamine profile revealed significantly increased levels of putrescine in T2D patients as compared to non-diabetic subjects. This finding could indicate an upregulation of the polyamine biosynthetic pathway, more specifically, the production of putrescine from ornithine by the biosynthetic enzyme ODC. In fact, putrescine levels have been shown to be elevated in kidney and liver in certain animal models of diabetes [16,35]. Given that the circulating levels of ornithine (the precursor of putrescine) are diminished in diabetic individuals, the higher putrescine levels cannot explained by a higher availability of substrate. In contrast, despite the lack of statistical significance, the serum levels of spermidine tended to be lower in the diabetic group. Since putrescine levels are higher in T2D, this suggests that the conversion of putrescine into spermidine (by spermidine synthase; (Figure 1)) may be impaired in T2D.

Correlation analyses further revealed a significant positive association of serum spermine and with circulating insulin levels and HOMA-IR. Because of the lack of correlation between spermine and glucose levels, these results suggest that spermine levels are more strongly associated with hyperinsulinemia than with insulin resistance in overweight or obese patients with metabolic disease. In pancreatic β cells, extranuclear polyamines mainly accumulate within secretory granules [30]. Whereas putrescine and spermidine appear to affect proinsulin biosynthesis, spermine may have a stimulatory role in long-term insulin release [31]. Thus, the positive correlation between circulating insulin and spermine could be explained by the fact that both molecules are likely co-secreted by pancreatic β cells. On the other hand, recent studies have reported beneficial effects of spermine administration in animal models with impaired glucose homeostasis. For instance, treatment with exogenous spermine has been shown to be effective in decreasing body weight and fasting glucose and improves glucose tolerance in diet-induced obese mice [24]. In addition, in rat adipocytes, spermine has been shown to enhance glucose transport and the conversion of glucose into triacylglycerols [36], and to increase the ability of insulin to bind to its own receptor [37]. Taken together, these studies suggest that spermine might modulate glucose homeostasis by acting as an endogenous insulin sensitizer when it is released into the circulation together with insulin; further studies are required to test this hypothesis.

In conclusion, in this study we demonstrate that serum putrescine levels are significantly increased in T2D and that they are positively correlated with HbA1c levels in a cohort of subjects with metabolic syndrome. In addition, we show that the serum levels of spermine are significantly correlated with insulin levels and HOMA-IR, suggesting the possible relationship of circulating spermine with elevated circulating insulin levels. In order to understand how T2D can influence polyamine metabolism (or vice versa), further studies are needed.

## Figures and Tables

**Table 1:** Baseline clinical and biochemical variables of the study subjects, according to the presence or absence of T2D.

	Non-Diabetic (*n* = 70)	T2D (*n* = 44)	*p* Value
Age, years	64.51 ± 4.46	63.75 ± 4.86	0.401
Gender, f (%)	34 (48.6)	22 (50.0)	-
Weight, kg	88.28 ± 12.83	92.29 ± 13.19	0.113
Waist, cm	112.57 ± 9.01	116.12 ± 8.66	0.039
BMI, mg/kg^2^	32.95 ± 3.38	34.23 ± 3.21	0.046
SBP, mm Hg	136.50 ± 13.92	141.82 ± 19.12	0.114
DBP, mm Hg	75.36 ± 9.51	74.91 ± 9.35	0.805
Glucose, mg/dL	103.43 ± 9.69	144.30 ± 34.86	<0.001
HbA1c, %	5.74 ± 0.29	7.15 ± 1.23	<0.001
Insulin, mUI/L	13.17 ± 7.05	20.38 ± 12.31	<0.001
HOMA-IR	3.42 ± 1.98	7.55 ± 5.70	<0.001
T2D treatment (insulin, metformin, other), *n* (%)	0 (0)	37 (84.1)	-
HDL-cholesterol, mg/dL	47.19 ± 11.19	46.11 ± 13.18	0.656
Triglycerides, mg/dL	181.93 ± 70.93	186.39 ± 138.78	0.844

Data are mean ± SD or n (%). *p* Values were calculated for the difference between study groups using either Student’s *t*-test or Mann–Whitney *U* test depending on the normality of the clinical variables. *p* < 0.05 was considered significant. BMI: body mass index; SBP: systolic blood pressure; DBP: diastolic blood pressure; HDL: high-density lipoprotein; HOMA-IR: homeostasis model assessment of insulin resistance; T2D: type 2 diabetes.

**Table 2:** Baseline serum polyamine levels of the study subjects, according to the presence or absence of T2D.

	Non-diabetic (*n* = 70)	T2D (*n* = 44)	*p* Value
Arginine, μg/mL	23.77 ± 4.54	21.99 ± 4.86	0.038
Ornithine, μg/mL	26.08 ± 5.59	23.47 ± 6.56	0.028
Putrescine, ng/mL	6.73 ± 2.34	7.54 ± 1.98	0.010
Spermidine, ng/mL	21.82 ± 9.23	21.09 ± 9.54	0.617
Spermine, ng/mL	5.65 ± 2.61	6.79 ± 3.44	0.067
Acetylputrescine, ng/mL	3.89 ± 1.33	3.86 ± 1.27	0.818
N^1^-Acetylspermidine, ng/mL	19.69 ± 6.39	19.78 ± 6.23	0.903
N^8^-Acetylspermidine, ng/mL	9.22 ± 2.15	8.88 ± 2.32	0.284
N^1^,N^8^-Diacetylspermidine, ng/mL	0.19 ± 0.12	0.24 ± 0.15	0.126
N^1^-Acetylspermine, ng/mL	0.60 ± 0.32	0.73 ± 0.36	0.053
N^1^,N^12^-Diacetylspermine, ng/mL	0.277 ± 0.22	0.29 ± 0.12	0.054

Data are mean ± SD or *n* (%). *p* values were calculated for the difference between study groups using either Student’s *t*-test or Mann–Whitney *U* test depending on the normality of the clinical variables. *p* < 0.05 was considered significant.

**Table 3:** Linear regression analysis with HbA1c as the dependent variable, and gender, age and putrescine as independent parameters.

	HbA1c (R = 0.350; R^2^ = 0.122)			
	β	Standard Error	95 % (CI)	*p* Value
Gender	−0.169	0.193	−0.552–0.214	0.383
Age (years)	−0.038	0.021	−0.080–0.004	0.076
Putrescine (ng/mL)	0.137	0.042	0.056–0.223	0.001

Dependent variable: HbA1c levels. Independent variables: gender (reference category: men (0) vs. women (1)); age (years); and serum putrescine (ng/mL).

**Table 4:** Linear regression analysis with HOMA-IR as the dependent variable, and gender, age, and spermine as independent parameters.

	HOMA-IR (R = 0.305; R^2^ = 0.093)			
	β	Standard Error	95 % (CI)	*p* Value
Gender	−0.046	0.815	−1.662–1.570	0.955
Age (years)	−0.178	0.090	−0.356–0.000	0.049
Spermine (ng/mL)	0.291	0.135	0.023–0.559	0.034

Dependent variable: HOMA-IR. Independent variables: gender (reference category: men (0) vs. women (1)); age (years); and serum spermine (ng/mL).

**Table 5:** Multiple logistic regression analysis: risk of T2D, adjusted for gender, age, BMI, and serum polyamine levels.

	OR	95% CI	*p* Value
Gender	1.190	0.499–2.838	0.694
Age (years)	0.974	0.886-–1.070	0.576
BMI (kg/m^2^)	1.098	0.968–1.246	0.144
Putrescine (ng/mL)	1.251	1.032–1.517	0.022
Spermidine (ng/mL)	0.952	0.904–1.003	0.066
Spermine (ng/mL)	1.205	1.026–1.416	0.023

Logistic regression analysis: risk (odds ratio [OR]) of T2D. Dependent variable: non-T2D (0) vs. T2D (1). Independent variables: gender (reference category: men (0) vs. women (1)); age (years); BMI (kg/m2); putrescine (ng/mL); spermidine (ng/mL); and spermine (ng/mL).

## Supplementary Materials

The following are available online at http://www.mdpi.com/2077-0383/8/1/71/s1. Supplementary Table S1: Multiple logistic regression analysis: risk of T2D, adjusted for gender, age, BMI, serum polyamine levels (putrescine, spermidine and spermine) and glucose (model 1); insulin (model 2); HOMA-IR (model 3).

## Abbreviations

BMI	body mass index
DBP	diastolic blood pressure
HDL	high-density lipoprotein
HOMA-IR	homeostasis model assessment of insulin resistance
ODC	ornithine decarboxylase
SBP	systolic blood pressure
SSAT	spermine/spermidine acetyltransferase
T2D	type 2 diabetes
UHPLC-MS/MS	ultra-high performance liquid chromatography tandem mass spectrometry.
