# Supplementary material for: Type 2 Diabetes Is Associated with a Different Pattern of Serum Polyamines: A Case–Control Study from the PREDIMED-Plus Trial
Source: J Clin Med. 2019 Jan 10;8(1):71. doi: 10.3390/jcm8010071 (PMC6352090; doi:10.3390/jcm8010071)
Supplement: Supplementary file 1 [file jcm-08-00071-s001.pdf]

Supplementary Table 1. Multiple logistic regression analysis: risk of T2D, adjusted for gender, age, BMI, serum polyamine levels (putrescine, spermidine and spermine) and glucose (model 1); insulin (model 2); HOMA-IR (model 3).

Model 1

	OR	P value	95% C.I.	
			Inferior	Superior
Gender	1.411	0.631	0.347	5.738
Age	1.050	0.498	0.912	1.209
BMI	1.040	0.701	0.850	1.274
Put	1.398	0.061	0.985	1.985
Spd	0.911	0.074	0.822	1.009
Spm	1.310	0.068	0.980	1.750
Glucose	1.226	0.000	1.123	1.337

Model 2

	OR	P value	95% C.I.	
			Inferior	Superior
Gender	1.298	0.574	0.523	3.225
Age	1.000	0.997	0.906	1.104
BMI	1.050	0.473	0.918	1.201
Put	1.229	0.040	1.009	1.498
Spd	0.960	0.147	0.909	1.014
Spm	1.151	0.111	0.968	1.369
Insulin	1.069	0.014	1.014	1.127

Model 3

	OR	P value	95% C.I.	
			Inferior	Superior
Gender	1.248	0.654	0.473	3.294
Age	1.027	0.612	0.925	1.141
BMI	1.013	0.861	0.875	1.173
Put	1.206	0.086	0.974	1.494
Spd	0.966	0.255	0.910	1.025
Spm	1.126	0.227	0.929	1.366
HOMA-IR	1.489	0.001	1.188	1.867

Logistic regression analysis: risk (odds ration [OR]) of T2D. Dependent variable: non-T2D (0) vs. T2D (1). Independent variables: gender (reference category: men(0) vs women (1)); age (years); BMI (kg/m2); putrescine (ng/ml); spermidine (ng/ml); spermine (ng/ml); and glucose (mg/dl; model 1); insulin (mUI/l; model 2); HOMA-IR (model 3).
